# A New Oral Testosterone Undecanoate Formulation Restores Testosterone to Normal Concentrations in Hypogonadal Men

**DOI:** 10.1210/clinem/dgaa238

**Published:** 2020-05-08

**Authors:** Ronald S Swerdloff, Christina Wang, William B White, Jed Kaminetsky, Marc C Gittelman, James A Longstreth, Robert E Dudley, Theodore M Danoff

**Affiliations:** 1 The Lundquist Institute and Harbor-UCLA Medical Center, Torrance, CA, US; 2 University of Connecticut School of Medicine, Farmington, CT, US; 3 Manhattan Medical Research, New York, NY, US; 4 UroMedix and South Florida Medical Research, Aventura, FL, US; 5 Longstreth and Associates, Mundelein, IL, US; 6 Clarus Therapeutics Inc., Northbrook, IL, US

## Abstract

**Context:**

A novel formulation of oral testosterone (T) undecanoate (TU) was evaluated in a phase 3 clinical trial.

**Objective:**

Determine efficacy, short-term safety, and alignment of new oral TU formulation with current US approval standards for T replacement therapy.

**Design:**

Randomized, active-controlled, open-label study.

**Setting and Patients:**

Academic and private clinical practice sites; enrolled patients were clinically hypogonadal men 18 to 65 years old.

**Methods:**

Patients were randomized 3:1 to oral TU, as prescribed (JATENZO^®^; *n* = 166) or a topical T product once daily (Axiron^®^; *n* = 56) for 3 to 4 months. Dose titration was based on average T levels (C_avg_) calculated from serial pharmacokinetic (PK) samples. T was assayed by liquid chromatography–mass spectrometry/mass spectrometry. Patients had 2 dose adjustment opportunities prior to final PK visit. Safety was assessed by standard clinical measures, including ambulatory blood pressure (BP).

**Results:**

87% of patients in both groups achieved mean T C_avg_ in the eugonadal range. Sodium fluoride-ethylenediamine tetra-acetate plasma T C_avg_ (mean ± standard deviation) for the oral TU group was 403 ± 128 ng/dL (~14 ± 4 nmol/L); serum T equivalent, ~489 ± 155 ng/dL (17 ± 5 nmol/L); and topical T, 391 ± 140 ng/dL (~14 ± 5 nmol/L). Modeling/simulation of T PK data demonstrated that dose titration based on a single blood sample 4 to 6 h after oral TU dose yielded efficacy (93%) equivalent to C_avg_-based titration (87%). Safety profiles were similar in both groups, but oral TU was associated with a mean increase in systolic BP of 3 to 5 mm Hg.

**Conclusion:**

A new oral TU formulation effectively restored T to mid-eugonadal levels in hypogonadal patients.

Male hypogonadism, or androgen deficiency, is diagnosed when unequivocally low serum testosterone (T) levels [typically <300 ng/dL (~10 nmol/L)] and consistent signs and symptoms are present (1). Regardless of the etiology, several signs and symptoms often can be managed with exogenous T replacement (2).

Testosterone replacement therapy (TRT) is administered by various delivery routes including transdermal gels and lotions; intramuscular and subcutaneous injections; surgically implanted pellets; dermal patches; intranasal gels; and oral capsules and tablets (methyltestosterone). Each of these delivery routes are associated with drawbacks well known to healthcare practitioners (HCP) and their patients [e.g., pain of injection, dermal irritation, T transference and liver toxicity (oral methyltestosterone)]. What has been absent from the HCP’s armamentarium of TRT products in the U. S. is an oral T formulation that meets current regulatory standards for safety and efficacy [e.g., FDA requires average serum T concentrations in the eugonadal range of 300–1000 ng/dL (10–35 nmol/L) for at least 75% of treated men with peak T concentrations largely below 1500 ng/dL (52 nmol/L)]. Historically, efforts to administer oral T have taken two primary paths: alkylation of T at the C-17 position to create T analogs that are resistant to first pass hepatic metabolism (exemplified by methyltestosterone dating to 1935 when first synthesized and used clinically by Ruzika) (3); or fatty acid esterification of T to create a T-ester [exemplified by T-undecanoate (TU)] that is absorbed via the intestinal lymphatic system thus bypassing the portal circulation (4). Oral methyltestosterone has been associated with serious hepatotoxicity such as cholestasis, peliosis hepatis, and hepatic adenocarcinoma (5-8) and therefore is rarely used in the clinical management of male hypogonadism. Conversely, while oral TU has not been associated with liver toxicity, an early oral TU formulation approved for use in many countries (but never in the U. S.) was highly influenced by dietary fat, thus leading to significant intra- and inter-patient variability in T response and questionable clinical utility (9, 10). Reformulation of this product to reduce the effect of dietary fat did not address the low TU content of the capsules thus resulting in the need to dose hypogonadal men with several capsules three or more times daily. Even then, reported serum T response would not result in average serum T levels in the normal range or meet current FDA efficacy standards (11). Accordingly, neither of these oral formulations has enjoyed widespread clinical use to treat T deficiency.

To address the absence of an oral TRT product that meets the rigor of current-day U. S. regulatory requirements for efficacy and safety, TU was formulated in a unique self-emulsifying drug delivery system that was initially evaluated in short-term clinical studies (12). In brief, the specific formulation we evaluated (encapsulated in soft gelatin capsules of various strengths) consisted of TU dissolved in a combination of lipids (principally long-chain fatty acids; e.g., oleic acid) and other solubilizers [e.g., borage seed oil (a rich source of C-20 fatty acids) and peppermint oil)] and a hydrophilic surfactant [hydrogenated castor oil (Cremophor^®^ RH 40)]. Formulations of this type enable the solubilization of highly lipophilic molecules like TU so that they may be absorbed after oral ingestion with food (high fat content not required) (13). Systemic delivery of oral TU occurs almost exclusively (>97%) via the intestinal lymphatic system, thereby bypassing the liver (4, 14). Once in the circulation, T is liberated from TU via the action of endogenous non-specific esterases. The undecanoic side chain (a C-11 fatty acid) is pharmacologically inert and metabolized by β-oxidation to acetyl coenzyme A (CoA) and, in the final step, propionyl CoA. Notably, during development of this new oral TU formulation, it became clear that enzymatic cleavage of T from TU can also occur during the standard laboratory processing of blood drawn from men treated with oral TU. The consequence of this post collection production of T was that assayed T values did not accurately reflect the actual circulating T concentration [i.e., they were artefactually high (15, 16)]. Therefore, in the pivotal clinical trial described herein, post-collection conversion of TU to T was minimized in men dosed with oral TU by assaying for T in plasma derived from blood collected into NaF-EDTA tubes that were held on ice prior to centrifugation (a process that halts all TU to T conversion). Because this sample handling approach is not typical in clinical practice, the dose titration algorithm utilized in the present trial (based on T measurements in NaF-EDTA plasma) was adapted for use with a single serum sample derived from blood collected into a standard plain collection tube (i.e., without added chemicals).

The present study was designed to assess the efficacy, based on T response and various patient-reported outcomes, and safety of a new oral TU formulation (JATENZO^®^) developed to treat male hypogonadism. In addition to standard safety assessments, we evaluated the potential impact of oral TU therapy on adrenal function and 24-hour ambulatory blood pressure. Finally, the effect of dietary fat content on T levels after oral TU administration was evaluated to determine if this was an important factor for T response.

## Materials and Methods

The Phase 3 clinical trial detailed herein was approved by a central or site-specific Institutional Review Board before study initiation at each clinical site and was conducted in accordance with the Declaration of Helsinki and/or all relevant federal regulations, including Good Clinical Practice guidelines. Written informed consent was obtained from trial participants before any study-related procedures were conducted (CLAR-15012; ClinicalTrials.gov identifier: NCT02722278).

### Study design

This trial was an open-label, repeat-dose, dose-titration study in hypogonadal men to assess the safety and efficacy of oral TU administered for approximately 3–4 months with a minimum of 90–105 days treatment prior to final PK visit. The Screening Phase was followed by a Titration Phase when there were two opportunities for dose-titration based on T C_avg_ calculated from a 24-hour serial pharmacokinetics (PK) evaluation, a 35-day Maintenance Phase, and an end-of-study PK visit. Eligible patients were randomly assigned in a 3:1 ratio to either oral TU or a 2% topical T solution. The starting dose for oral TU was 237 mg TU (150 mg of unesterified T equivalents), twice-daily (BID) which was administered immediately prior to a breakfast and dinner meal, approximately 12 hours apart. The starting dose for topical T was 60 mg once daily (QD) in the AM. For dose titrations, serial PK samples over 24 hours were obtained around Days 21 and 56 to determine C_avg_ for dose titrations that occurred about 14 days after the PK visits. Based on the T C_avg_, oral TU doses could be up-titrated sequentially to 316 mg (200 mg T equivalents) and then 396 mg (250 mg T equivalents), or down-titrated to 198 mg (125 mg T equivalents) and then 158 mg (100 mg T equivalents) BID. Similarly, topical T doses could remain unchanged or be increased to 90 or 120 mg or decreased to 30 mg. The primary efficacy endpoint was based on the T C_avg_ at the final PK visit. A cosyntropin [ACTH (1-23)] stimulation test was conducted on a subset of patients (N = 24 on oral TU and N = 8 on topical T).

### Patient population

Eligible patients were men aged 18–65 years, body mass index <38 kg/m^2^, with hypogonadism as defined by consistently low morning serum total T <300 ng/dL (blood samples collected between 6:00 and 10:00 AM on 2 separate days approximately 7 days apart) and a history of signs and/or symptoms consistent with hypogonadism. Patients were naïve to androgen-replacement therapy or had a complete washout period of previous androgen replacement therapies [2 wks for oral, topical (gel or patch), intranasal or buccal T; 4 wks for short-acting i.m. T (e.g., T-enanthate, T-cypionate); 20 wks for i.m. TU; and 6 months for s.c. T-pellets]. Patients were excluded if they had significant uncontrolled intercurrent disease of any type, hematocrit (Hct) <35% or >48%, history of polycythemia, untreated, severe obstructive sleep apnea, abnormal digital rectal exam, prostate-specific antigen (PSA) >4.0 ng/mL, International Prostate Symptom Score >19 or history of prostate cancer. Prohibited medications included those that could affect T levels, T metabolism, or levels of T metabolites (e.g., antiandrogens, 5-alpha-reductase inhibitors, estrogens, long-acting opioid analgesics or human growth hormone). Patients participating in a cosyntropin stimulation sub-study were not allowed treatment with corticosteroids (oral or inhaled) and were excluded if they had a pituitary abnormality (e. g., hypopituitarism, post-surgery, post-radiotherapy, or history of abnormalities on imaging such as an adenoma).

### Primary and other secondary endpoints

The primary efficacy endpoint was mean T concentration (C_avg_) after two dose-adjustment cycles with the objective being to demonstrate that at least 75% (with a lower 95% CI of 65%) of patients treated with oral TU (JATENZO^®^) achieved a T C_avg_ in the eugonadal range of 252 to 907 ng/dL (9 to 31 nmol/L) for blood collected in NaF-EDTA tubes. A topical T solution (i.e., Axiron^®^) was used as the active T comparator.

Peak T concentrations (C_max_) were also measured over 24 hours at several points during the study and formed the basis of a secondary efficacy assessment based on FDA criteria. Specifically, T-replacement products must yield a C_max_ response that is in close alignment with the following targets: ≥85% of patients with a C_max_ < 1500 ng/dl (52 nmol/L); ≤ 5% of patients with C_max_ between 1800–2500 ng/dL (62–87 nmol/L); and no patients with a C_max_ > 2500 ng/dL) (87 nmol/L). These C_max_ categories were ostensibly based on the upper eugonadal limit in serum of 1000 ng/dL (35 nmol/L). However, the eugonadal T range in NaF-EDTA plasma when assayed by LC/MS-MS only extended to 907 ng/dL; therefore, C_max_ was also evaluated (*post hoc*) in light of a second set of C_max_ categories based on T assays in this matrix, namely, ≥ 85% of patients with a C_max_ ≤ 1361 ng/dL (47 nmol/L); ≤ 5% of patients with C_max_ between 1633–2268 ng/dL (57–79 nmol/l); and no patients with a C_max_ > 2268 ng/dL (79 nmol/L).

### Pharmacokinetic and efficacy assessments

The efficacy endpoints of the trial were based on PK of NaF-EDTA plasma T in patients treated with oral TU. Blood samples for total T, dihydrotestosterone (DHT), and estradiol were collected in plain tubes that were allowed to clot for 30 minutes at room temperature (for serum) and NaF-EDTA-containing tubes (BD vacutainer) that were placed on ice for 30 minutes (for NaF-EDTA plasma) prior to centrifugation on study PK days (namely, prior to the 2 dose titration visits and final study visit). Samples from patients in the oral TU group were obtained at -30 minutes and 0, 2, 4, 6, 9, and 12 hours after AM dose and 0 (i.e.,12 hours after AM dose), 2, 4, 6, 9, and 12 hours after PM dose. For patients in the topical T group blood samples were collected at -30 minutes and 0, 2, 4, 6, 9, 12, 14, 16, 18, 21, and 24 hours after AM dose.

Total T and DHT in NaF-EDTA plasma samples were measured using a previously reported validated liquid chromatography tandem-mass spectrometry (LC-MS/MS) assay (17). Serum total T concentrations for screening and comparative results for the final PK visit were determined using validated measurements by LC-MS/MS as previously described (12, 13). Estradiol was assayed using a validated LC-MS/MS assay (18) in serum using samples collected at the last study visit.

The concentrations of total T and DHT were analyzed to derive the PK parameters of C_max_, time to maximal T concentration (T_max_), area under the plasma concentration-time curve (AUC) and C_avg_ for both the morning and evening dosing intervals (oral TU only), as well as the entire 24-hour period (oral TU and topical T). The concentrations of estradiol collected at the final visit were similarly analyzed. Free T concentrations were calculated using the Vermeulen formula (19) based on T, sex hormone binding globulin (SHBG), and albumin concentrations.

The Psychosexual Daily Questionnaire (PDQ) (20) was used to assess sexual function and mood changes. Patients were asked to complete the questionnaire every day for 7 consecutive days before Day 1 and the last study day (end of study). Each domain of the PDQ (sexual desire, enjoyment and performance, mood, and sexual activity score) was evaluated.

### Safety assessments

Safety was assessed by recording serious adverse events (SAEs), treatment-emergent adverse events (TEAEs), and routine clinical chemistry and hematology laboratory measurements (particularly hematocrit, PSA and lipid profile). Prostate symptoms were assessed using the International Prostate Symptom Score (I-PSS) (21). Vital signs were measured following published guidelines for accurate measurement of BP (22) in triplicate in the office by an automated oscillometric device. The average of the 3 office measurements was used for data analyses. In addition, 24-hour blood pressure (BP) and heart rate values were acquired every 20 minutes through ambulatory BP monitoring (ABPM) (Spacelabs, Inc, Redmond, WA) performed on the day before the baseline visit and again 1–3 days prior to the final PK Visit.

### Data analyses

#### Efficacy / pharmacokinetic analyses.

Efficacy was assessed based on the percentage of treated patients whose T C_avg_ was in the eugonadal range. The T C_avg_ was separately summarized for those receiving oral TU and topical T, respectively, without a formal comparison between the treatment groups. The C_avg_ was calculated by non-compartmental PK methods using actual sample collection times relative to dosing using a modified intent-to-treat population (mITT). In the efficacy analysis, patients who dropped out prior to final study day due to a possible treatment-related cause (e.g., an AE), were counted as treatment failures, while patients who dropped out for other causes (e.g., site closure not related to study conduct) had their T C_avg_ imputed using last observation carried forward (LOCF) methodology. A 95% Clopper-Pearson binomial confidence interval (CI) for the proportion was reported along with the estimated proportion.

Standard descriptive PK for the oral TU and topical T, as calculated using non-compartmental methods, were presented for total T, free T (calculated), DHT and estradiol. In addition, changes from the pretreatment baseline to end-of-study were presented for the endogenous molecules LH, FSH, and SHBG.

#### Psychosexual daily questionnaire analyses.

 The Psychosexual Daily Questionnaire weekly average subscale scores were computed using the daily scores collected during the visit period as previously described (20), but only if the daily questionnaire had been completed on at least 3 of the 7 consecutive days in the period.

#### Cosyntropin stimulation test.

To ensure that the new oral TU formulation did not cause a reduction in cortisol levels, a cosyntropin [ACTH (1-23)] stimulation sub-study was conducted at a select number of centers in this study to evaluate the effect of testosterone on the hypothalamic-pituitary-adrenal axis and determine if chronic oral TU or topical T treatment suppressed cortisol secretion. At the initial study visit (before administration of oral TU or topical T) and at the last study visit study (i.e., the day after collection of final 24-hr PK samples), a baseline blood sample was drawn followed by intravenous injection of 0.25 mg cosyntropin. Blood samples for serum cortisol assay by liquid chromatography tandem mass spectrometry (LC-MS/MS) were subsequently collected 30 and 60 minutes after the injection. For each treatment group, the adrenal response to cosyntropin was evaluated by estimating the proportion of patients whose maximum cortisol value was normal (18 mcg/dL) at the pre-treatment visit and again at the final.

#### Evaluation of food effect.

This sub-study was conducted only in oral TU patients. At the start of the study, patients were offered several meal choices during confinement, without disclosing the nutritional content of the various meal options. The patients were asked to select the meal that best reflected what they would typically eat for breakfast and dinner (meals at which oral TU dosing occurred). After the meal selection, the same meal was served at all PK visits during the study. The meal choices had defined amounts of fat (15 g, 30 g, or 45 g). The unconsumed parts of the meal were recorded to estimate actual fat intake. In this way, a more real-world setting for oral TU use was integrated into the design of the study.

The food-effect analysis included all patients on each PK day for which the subject was evaluable for testosterone concentrations for AUC_AM_, AUC_PM_, and AUC_24_. The AM- and PM-specific PK parameters were examined for a dependence on AM or PM meal type, respectively. The comparisons were examined using dose normalized AUC_x_ values. Pearson’s correlation coefficients between the fat intake and the dose normalized AUC_x_ values were calculated.

The distribution of final titrated dose by the various meal compositions at earlier study visits were examined to ascertain if meal type played a significant role in the dose to which patients were titrated. This was done using a cross-tabulation and a Pearson’s chi-square association test. Dose distributions were examined for the breakfast and dinner meals separately.

#### PK simulation/modeling and concordance analyses.

Two approaches were taken to determine the optimal time for the assessment of circulating T on the basis of a single blood sample after the morning oral TU dose in order to provide dosing guidance for oral TU in real world clinical settings where T response to TRT is determined from a single blood sample. First, extensive simulation and modeling of PK data was used to identify a discrete blood sampling range (e.g., 4 to 6 hours after oral TU) that would consistently yield a T value in close agreement with the actual T C_avg_ based on serial PK samples. The population PK of T following oral TU dosing were modeled with a 1-compartment model with first order absorption following a time lag, a central volume of distribution, and first order clearance. Inter-subject and inter-occasion variability were incorporated into the time-lag, absorption, volume and clearance parameters. Testing of each candidate status sample collection time and choice of cut-off thresholds utilized 1000 simulated patients run through the protocol- designated TU dose titration schedule and maintenance treatment period. The aforementioned sources of inter-occasion variability introduced random variability among the multiple T concentration assessments (i.e., each titration visit and end of study) made for each simulated subject. Second, concordance analysis was performed to identify the best post-dose T assay time-point to guide any necessary dose-adjustment in oral TU patients. Concordance is defined herein to describe the extent of agreement between a decision to adjust the oral TU dose (up or down) when a single circulating T concentration in remains in the hypogonadal range [i.e., <252 ng/dl (9 nmol/L) for this study] or supraphysiological range [i.e., >907 ng/dL (31 nmol/L) for this study] and the desired outcome of that decision (i.e., a circulating T level in the eugonadal range) is achieved. Although a predefined concordance result was not established for this study, the goal was to demonstrate as high a total concordance as possible (e.g., at least 85%).

Derivation of a Conversion Factor to Allow use of Serum for T measurements after Oral TU Administration

In this study, plasma samples were collected in NaF-EDTA tubes to minimize *ex-vivo* conversion of TU to T. However, in clinical practice serum T is routinely collected from blood drawn into in plain tubes. Because the post-collection conversion rate of TU to T is higher in serum (blood collected in plain tubes) compared to plasma (blood collected in NaF-EDTA tubes) (15, 16), a Phase 1 study (CLAR-18019, ClinicalTrials.gov identifier: NCT03973840) provided data for calculation of the conversion factor between T levels measured in blood collected in NaF-EDTA tubes and plain tubes from men who received oral TU.

## Results

### Subject disposition and baseline characteristics

**Figure 1.**
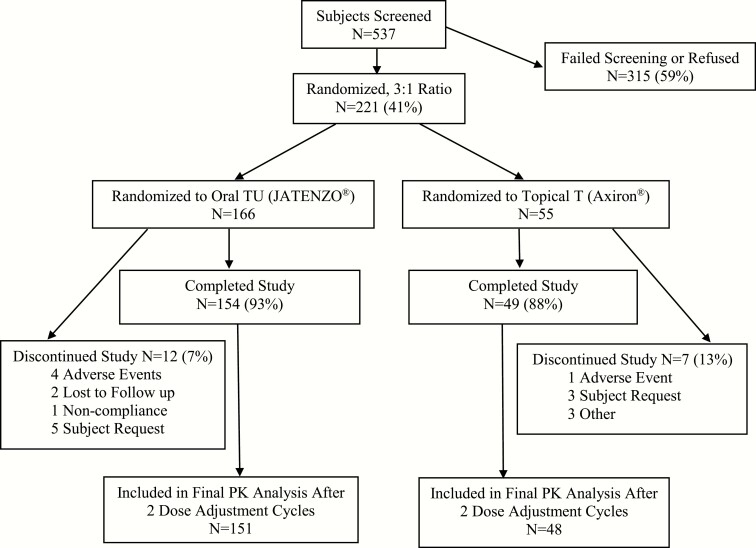
Overall subject disposition by treatment group for intent-to-treat pharmacokinetics (PK) populations.

A total of 221 eligible patients were randomized and received treatment in a 3:1 ratio to oral TU (JATENZO^®^; N = 166) or topical T (Axiron^®^; N = 55). Approximately 92% of the patients who were randomized to oral TU completed the study compared to 88% in the topical T arm. Fig. 1 summarizes the overall subject disposition by treatment group for the intent-to-treat population. Demographics and baseline characteristics were similar between the two treatment arms as summarized in Table 1.

**Table 1.** Demographics and baseline characteristics at baseline of intent-to-treat hypogonadal male population

Characteristic	Oral TU (N = 166)	Topical T (N = 56)
Age (years)		
Mean (SD)	51.6 (9.08)	53.4 (7.86)
Race, n (%)		
American Indian or Alaska Native	0	1 (1.8)
Asian	3 (1.8)	2 (3.6)
Black or African American	29 (17.5)	11 (19.6)
White	133 (80.1)	42 (75.0)
Other	1 (0.6)	0
Height (cm)		
Mean (SD)	178.4 (6.81)	178.4 (7.61)
Weight (kg)		
Mean (SD)	101.4 (15.75)	98.2 (14.24)
Prior testosterone therapy, n (%)	166 (100)	56 (100)
BMI (kg/m^2^) ^*^		
Mean (SD)	31.8 (4.16)	30.9 (4.13)
Median	32.2	30.6
Minimum, maximum	17, 38	21, 38
BMI categories, n (%)		
Under weight: < 18.50 (kg/m^2^)	1 (0.6)	0
Normal weight: 18.50–24.99 (kg/m^2^)	7 (4.2)	4 (7.1)
Overweight: 25.00–29.99 (kg/m^2^)	50 (30.1)	20 (35.7)
Obese: ≥ 30.00 (kg/m^2^)	108 (65.1)	32 (57.1)
Blood pressure (mm Hg)		
Systolic pressure (SD)	126.9 (11.47)	123.5 (13.18)
Diastolic pressure (SD)	79.1 (7.84)	77.1 (8.03)
History of hypertension, n (%)		
Yes	87 (52.4)	26 (46.4)
No	79 (47.6)	30 (53.6)

Abbreviations: BMI, body mass index; ITT, intention-to-treat; SD, standard deviation; TU, testosterone undecanoate.

### Efficacy results

Primary and secondary efficacy results are summarized in Table 2. Based on T results obtained at the final PK visit of the study, 87.3% of patients in the oral TU group had T C_avg_ values in the eugonadal range, with a mean ± SD value of 403 ± 128 ng/dL (14 ± 4 nmol/L) based on T assay of NaF-EDTA plasma. When expressed in terms of approximate equivalent *serum* T concentration (15), the mean ± SD value was 489 ± 155 ng/dL (17 ± 5 nmol/L). Of those patients dosed with topical T, primary efficacy identical to oral TU was observed (i.e., 87.3%) based on final visit T assays. Sensitivity analyses [i.e., last-observation-carry-forward (LOCF), multiple imputation and imputation from baseline] were performed to assess the impact of missing T data at the final PK visit for oral TU patients. All three analyses resulted in estimates of the percentage of patients in the eugonadal T range of 86 to 90%.

**Table 2.** Percentage of patients with testosterone (T) C_avg_ values in the eugonadal range at end of study for primary analysis (modified ITT population)

	FDA Target	Oral TU	Topical T
T concentration			
Patients, N		166	55
C_avg_ range, 252 ng/dL ≤ C_avg_ ≤ 907 ng/dL,% (n)^a^		87.3% (145))	87.3% 48))
95% CI		81.3%, 92.0%	75.5%, 94.7%
C_avg_ mean (SD) ng/dL (NaF-EDTA plasma)		402.5 (127.7)	390.6 (139.9)
95% CI		379.7, 422.7	352.8, 428.5
C_avg_ mean (SD) ng/dL (serum equivalent)		488.7 (154.5)	474.2 (169.8)
95% CI		461.0, 513.2	428.3, 520.2
Serum testosterone			
Patients, N		151	48
C_max_ range, % (n)			
≤1500 ng/dL	≥85%	90.7% (137)	97.9% (47)
>1800–2500 ng/dL	≤5%	2.0% (3)	2.1% (1)
>2500 ng/dL	0	2.0% (3)^c^	0
NaF-EDTA Plasma			
Patients, N		151	48
T C_max_ Range^d^, % (n)			
≤1361 ng/dL	na	82.8% (125)	97.9% (47)
>1633–2268 ng/dL	na	3.3% (5)	2.1% (1)
>2268 ng/dL	na	2.6% (4)^c^	0

Abbreviations: C_avg_, T average observed concentration over 24 h; C_max_, maximum observed concentration over 24 h; ITT, intention-to-treat; TU, testosterone undecanoate; FDA, Food and Drug Administration.

^a^Eugonadal range for T measured by LC/MS-MS in NaF-EDTA plasma; serum equivalent = 306-1100 ng/dL.

^b^Eight patients had C_max_ values >1500 to ≤1800 ng/dL.

^c^All 3 patients had C_max_ values indicative of sample contamination.

^d^Post hoc analysis based on upper limit of eugonadal range for T assayed in NaF-EDTA plasma. FDA has not established T C_max_ targets for NaF-EDTA plasma matrix.

eThree patients had C_max_ values indicative of sample contamination

At the end of the study, values of T C_max_ ≤1500 ng/dL were observed for 90.7% of patients in the oral TU group and 97.9% of patients in the topical T group. None of the patients treated with oral TU experienced a C_max_ value >2500 ng/dL except for three spurious and transient C_max_ excursions above 2500 ng/dL that were determined to be the result of external contamination of the 2-hour post-dose plasma samples at a single study site where plasma samples were being prepared from topical T patients at the same time as samples from those dosed with oral TU. If a blood sample collected from an oral TU-treated subject was contaminated with exogenous T, the expectation was that the T concentration in the sample would be enhanced but the DHT concentration would not be. And this was found to be true. The DHT/T molar ratios for the three suspect samples where all were between 0.0439 and 0.0602, values that were less than half the mean DHT/T ratio (0.1484) observed in 2-hour post-dose samples of the other oral TU-treated subjects.

### Effects on sexual function and mood

**Figure 2.**
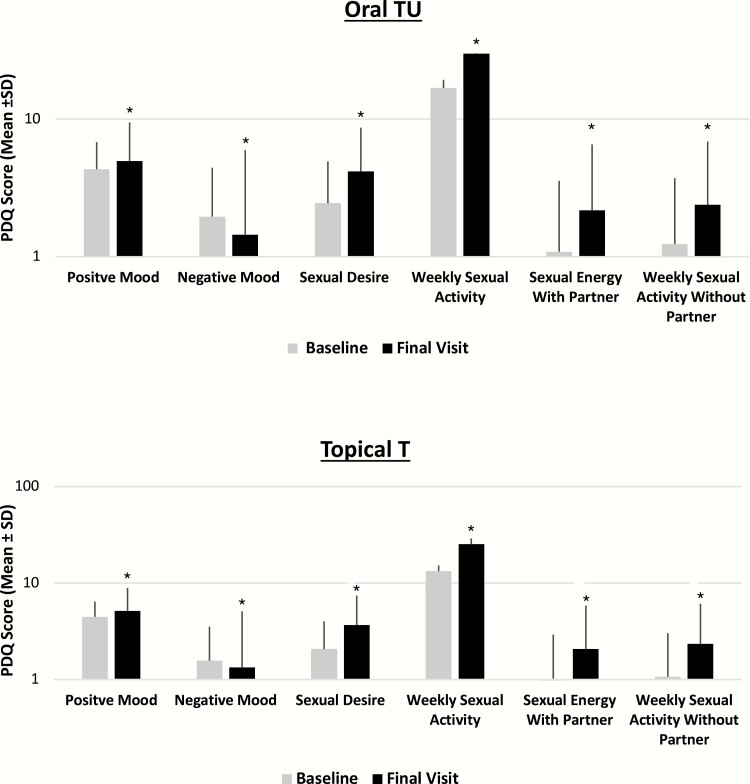
Effects of oral TU and topical T on mean change from baseline in Psychosexual Daily Questionnaire data at end of study (all T-treated patients). *Statistically signficant difference from baseline (*P* < 0.0001).

As illustrated in Fig. 2, both the oral TU and topical T groups demonstrated significant improvements from baseline (p < 0.0001) in each of the psychosexual parameters, with mean increases from baseline noted for sexual desire, sexual enjoyment with and without a partner, sexual activity, satisfaction with erection duration, % full erection, and positive mood and mean decreases noted for negative mood. No significant differences in magnitude of change from baseline between the treatment groups (p > 0.05 for all comparisons) were observed for any of the Psychosexual Daily Questionnaire parameters.

### Oral TU dose titration and pharmacokinetics

The average dose of oral TU increased with each titration cycle. Overall, there were more up-titrations than down-titrations. Among the 155 oral TU patients who completed the study, approximately 72% were up-titrated from the initial dose (32% to 316 mg and 40% to 396 mg TU, BID), 26% remained at their initial oral dose of 237 mg TU, BID, and 3% were down-titrated (237 to 198 mg TU, BID). Among the 49 topical T patients who completed the study, approximately 45% required no titration from the initial dose of 60 mg QD, while the remaining patients were titrated up in dose.

**Figure 3.**
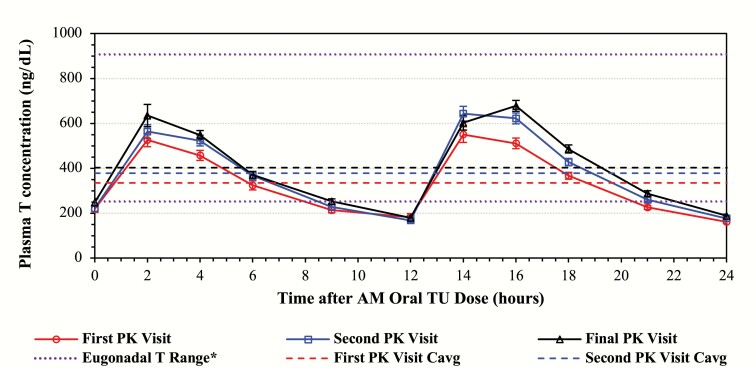
Mean (±standard error) concentration-time profiles for NaF-EDTA plasma total T in patients treated with oral TU at the first, second, and final pharmacokinetics (PK) visit. Values in graphs can be converted to approximate serum T equivalents by multiplying by 1.214 (see text for detail). *As measured in NaF-EDTA plasma.

The average dose of oral TU progressively increased during the titration process from the starting dose of 237 mg oral TU, BID to 287 mg TU in response to dose adjustments made at the first titration visit to 325.1 mg TU after dose adjustments were made at the second (and final) titration step. Upward adjustments to oral TU dose were consistent with the increases in the mean T concentration-time profiles across the three different visits. Mean concentration-time profile for NaF-EDTA plasma total T at the PK visits is shown in Fig. 3 for the oral TU patients.

When the T results of all patients at a particular visit were combined, regardless of the dose of oral TU they received, the mean peak T over 24 hrs. (C_max24_) for oral TU patients ranged from approximately 800 ng/dL at the first dose titration to 1000 ng/dL at the end of study and occurred at median times of approximately 2–4 hours following the AM dose, and approximately 4 hours following the PM dose. The mean NaF-EDTA plasma (approximate equivalent serum T) C_avg24_ values ranged from 335 (407) ng/dL at the first titration to 403(489) ng/dL at the final PK visit. The coefficients of variation (CVs) for C_avg24_ decreased as the visits progressed through the study (46.7% to 37.7% to 31.7%), as would be expected since the titration process was designed to down titrate those patients with high C_avg24_ values and up titrate patients with low C_avg24_ values, thus progressively narrowing the distribution of C_avg24_ values with each titration step. The outcome of this process is depicted graphically in Fig. 3. The mean plasma total T PK parameters at the end of study are summarized by treatment for all doses combined in Table 3.

**Table 3.** Summary of oral TU and topical-T NaF-EDTA plasma total testosterone (T) pharmacokinetic parameters by treatment

			Oral TU (All Doses)			Topical-T (All Doses)		
Visit	PK Parameter	Units	N	Mean	SD	N	Mean	SD
Baseline	Plasma T	ng/dL	165	206.8	80.72	54	202.7	91.82
Final day	C_avg24_^a^	ng/dL	151	402.5^d^	127.72	48	383.0	131.36
	C_max24_	ng/dL	151	1008.3	581.04	48	664.0	319.23
	T_max-__am_^b,c^	h	155	3.87	(0.00, 12.08)	48	4.01	(0.00, 24.00)
	T_max__-pm_^b^	h	151	16.00	(12.00, 24.02)			

Abbreviations: AM, morning; C_avg24_, time-weighted average plasma T concentration AM and PM doses combined; C_max24_, mximum observed T concentration AM and PM doses combined; C_max-__AM_, time-weighted average T concentration over the daytime dosing interval following the AM dose; C_max-__PM_, time-weighted average concentration over the daytime dosing interval following the PM dose; PK, pharmacokinetic; PM, evening; SD, standard deviation; T_max-__AM_/T_max-__PM_, Time to C_max-__AM_/C_max-__PM_; TU, testosterone undecanoate.

^a^C_avg24_ calculated using actual sample collection times.

^b^T_max_ values are median (range) after AM oral TU dose; Tmax_PM_ values are median (range) after AM oral dose. Mean (range): 12 h = T_max_ relative to PM oral TU dose.

^c^Topical T_max_ is relative to the AM dose since it was applied once daily each morning.

^d^Measured in NaF-EDTA plasma. Approximate serum T value = 489 ± 155 ng/dL.

### Single-sample dose-adjustment paradigm for oral TU

**Figure 4.**
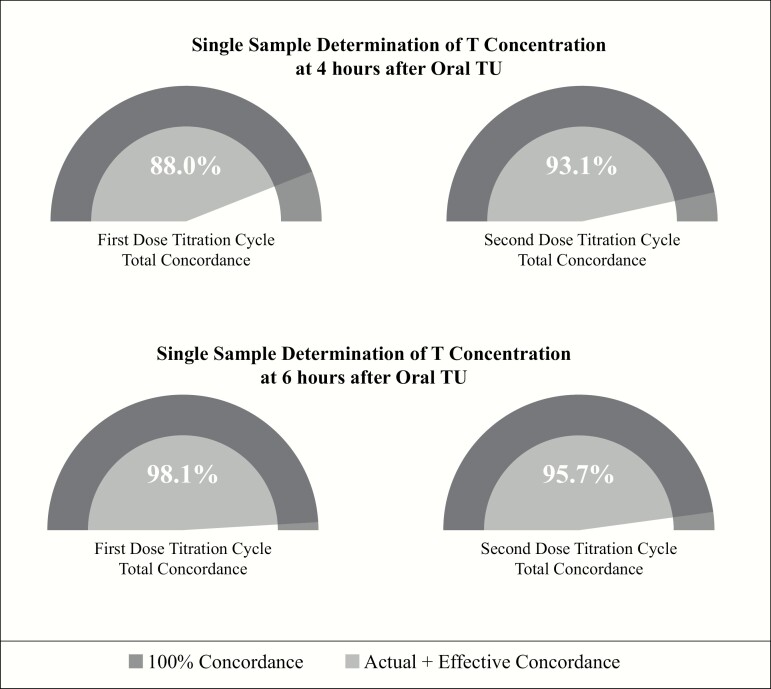
Concordance between decision to adjust oral TU dose on basis of single sample determination of circulating T concentration at 4 and 6 h after the morning oral TU dose and outcome of decision for first and second dose-titration cycles.

Pharmacokinetic (PK) modeling and simulation results for circulating T confirmed that dose titration decisions based on a single blood sample taken 3–5 hours or at 4 hours after the morning oral TU dose was an effective means to guide dose adjust to achieve/maintain T concentrations in the eugonadal range. As shown in Table 4 regardless of the three measures used to determine the need to adjust the oral TU dose (i.e., Cavg based on serial PK blood sampling, a single blood sample 4 hours after the morning dose or a single sample taken any time between 3 to 5 hours after oral TU), efficacy was high with 95% of patients achieving a mean T concentration in the eugonadal range and <5% of patients with a mean T level below normal. Concordance analysis of the results from the first and second dose-titration visits are shown in Fig. 4 [tabular concordance data are available in Dryad Digital Repository (23)]. These results demonstrated that for the first and second PK visits, total concordance was 88% and 93%, respectively, when a single blood sample for T assay was collected 4 hours after the oral TU dose. When total concordance was analyzed on the basis of a single T concentration at 6 hours after oral TU at the first and second PK visit, total concordance was 98 and 96%, respectively.

**Table 4.** Simulation results evaluating mean T concentration (C_avg_) vs time of single sample collection after oral TU as surrogate for true C_avg_

Estimated % of Patients with C_avg_ Within T Interval (95% CI) on Final PK Visit			
	< 252 ng/dL	252–907 ng/dL	> 907 ng/dL
Target at final PK visit		≥75%	
C_avg_-based titration schemes	3.4 (0.0-8.5)	94.8 (88.9-99.0)	1.8 (0.0-4.0)
Single draw status sample at defined time point (C_4_)	4.6 (1.5-8.7)	94.4 (89.8-98.0)	1.0 (0.0-3.1)
Single draw status sample in C_3-5_ window	4.7 (1.0-10.0)	94.3 (89.0-98.5)	1.0 (0.0-3.0)

Abbreviations: T, testosterone; C_3-5_, T concentration 3 to 5 h after morning dose; C_4_, T concentration 4 h after morning dose; C_avg_, average observed concentration over 24 h; CI, confidence interval; C_max_, maximum T concentration.

Derivation of a Conversion Factor to Permit use of Serum v. NaF-EDTA Plasma T to Guide Dose Adjustments in Men Treated with Oral TU in a Clinical Setting

As noted previously, in order to monitor T levels in patients receiving oral TU using standard blood collection techniques (i.e., plain tube for serum T assay), it was necessary to derive a conversion factor which accounted for TU-to-T conversion in serum from blood that had clotted at room temperature v. NaF-EDTA blood collection tubes held on ice that prevented this conversion (i.e., blood collection method as used in current Phase 3 clinical trial). We have shown previously that the amount of T generated due to TU-to-T conversion is, in part, a function of TU concentration and that NaF directly decreased T levels assayed by LC/MS-MS when blood was collected into NaF containing tubes (15). Thus, we were able to calculate a conversion factor based on the regression equations or the estimated TU concentration 6 hours post-dose, namely, 52 ng/mL (obtained from a non-published study; ClinicalTrials.gov Identifier: NCT01403116). To convert a T concentration measured in NaF-EDTA plasma to an equivalent T concentration measured at C_6_ in serum required multiplying the NaF-EDTA plasma T concentration by 1.214. This overall correction factor is the product of three independent factors: 0.999 (to account for the small amount of overestimation in the NaF-EDTA containing tube) x 1.043 (to account for the overestimation of T that would occur in the plain tube due to TU to T conversion) x 1.166 [to account for a NaF matrix effect (NaF-EDTA plasma v. serum)] on T measurement (15). Thus, it was possible to obtain a close estimation of the equivalent serum T concentration in samples collected 6 hours post-dose when the T concentration was measured in NaF-EDTA plasma. To test the applicability of this conversion factor (derived from the N = 13 blood collection study), it was applied to NaF-EDTA plasma T data generated from oral TU patients at their final PK visit. At that visit, duplicate PK samples were collected in NaF-EDTA and plain tubes so T concentrations could be measured in both. Applying the conversion factor resulted in a mean (95% CI) error of only 3.1% (0.4%, 5.8%)—too small to impact essentially any dose titration decision. Furthermore, this error is substantially less than the 15% error allowed for most clinical assays (24). Finally, this conversion factor was integrated (invisibly) into product labeling for the new oral TU formulation such that health care providers can assess T response and make dose-titration decisions based on serum T derived from blood collected into standard plain tubes (i.e., devoid of any chemicals).

### Food effect

**Figure 5.**
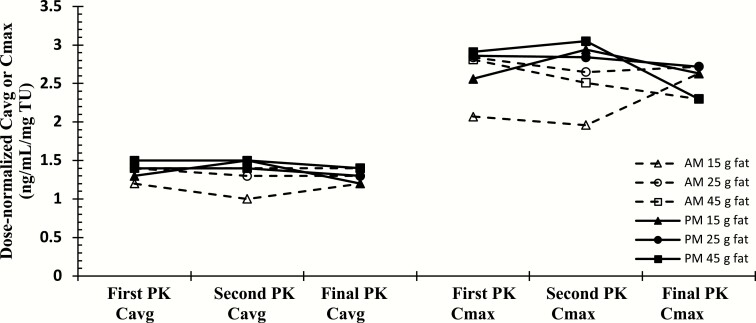
Dose-normalized mean average T concentration (C_avg_) at PK visits for oral TU patients (stratified by mean dietary fat content).

There was no clinically significant difference in dose-normalized T C_avg_ among the meal types, indicating that dose titration based on post-AM blood samples was not significantly impacted by the AM meal fat composition between 15 to 45 g fat (see Fig. 5). There was also no significant effect of food on peak T (C_max_) concentrations. These data demonstrate the reproducibility of PK responses of patients on a particular diet (i.e., with respect to fat content) over the PK visits, regardless of their oral TU dose. Dose normalization compensates for any dose titration changes between PK visits. The PK patterns were similar with both the AM and PM doses and even though the high fat and very low fat patients had the most variability in the between visit PK values, they were not substantially different between visits, nor substantially different from patients on diets with other fat content.

### Changes in calculated free T, SHBG, Estradiol, DHT, LH and FSH after oral TU

**Figure 6.**
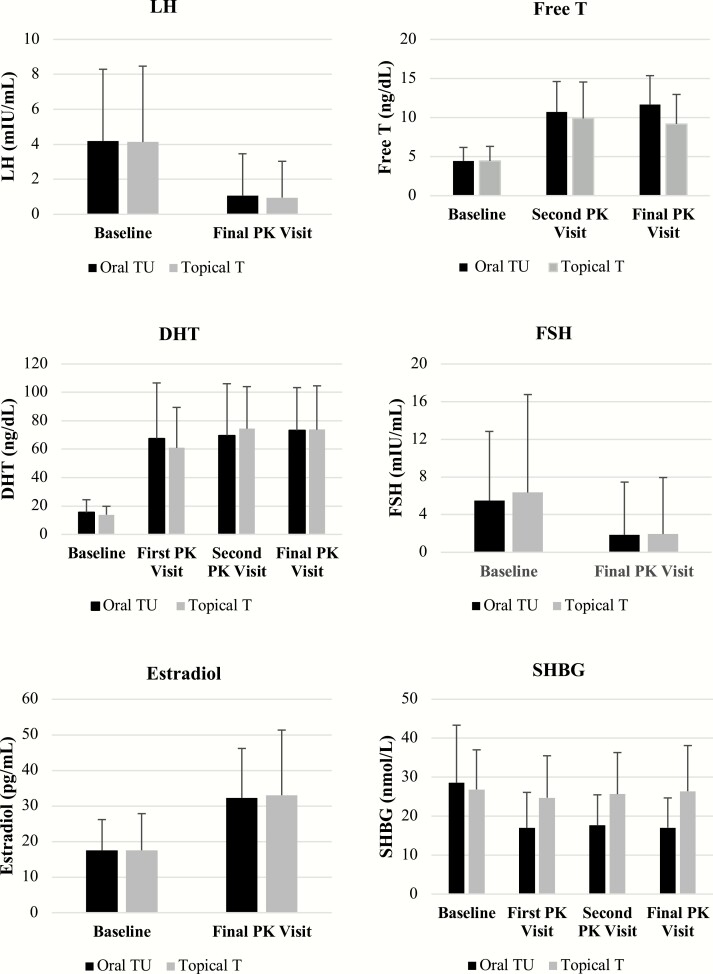
Effect of oral TU and topical T on LH, Free T, DHT, FSH, estradiol, and SHBG over course of T therapy.

Effects of oral TU and topical Ton calculated free T, DHT, estradiol (E_2_), LH, FSH and SHBG are depicted in Fig. 6. As expected in both treatment groups, T administration caused significant elevations from baseline in free T, DHT, and estradiol and decreases in, LH, FSH and SHBG. The magnitude of effects observed in oral TU patients paralleled those seen in patients treated with topical T and the differences in responses between the treatment groups was not statistically different. However, there was a trend toward higher free T concentrations in oral TU patients compared to topical T patients and a greater mean decline in SHBG in oral TU patients. The greater increase in free T from baseline for the oral TU group was partly a function of a 36% decrease in mean SHBG from 28.6 ± 14.7(SD) to 17.0 ±7.6 nmol/L in the oral TU group compared to essentially no change in the topical T group from 26.8 ± 10 to 26.4 ± 11.7 nmol/L. However, both baseline and final mean SHBG concentrations remained within the normal range for eugonadal men (10.8 to 46.6 nmol/L) at the final study visit in both groups.

Over the course of the study, mean estradiol levels increased to slightly above the upper end of the eugonadal range in both treatment groups [oral TU: 32 ± 14 pg/mL (117 ± 51 pmol/L) and topical T: 33 ± 18 pg/mL (121 ± 66 pmol/L]. Plasma DHT concentrations for the oral TU- and topical T treated patients were essentially identical at all PK visits and at the final visit [73 ng/dL (2.5 nmol/L) were slightly above the normal upper limit of 65 ng/dL (2.2 nmol/L). Mean change from baseline in the serum concentrations of LH and FSH at end of study (AM pre-dose concentration) in the oral TU and topical T patients showed an approximately 70% decrease from mean baseline values.

### Safety results

The overall incidence of treatment-emergent adverse events (TEAEs) considered related to study drug occurred in 18.7% of patients in the oral TU group and in 14.5% of the topical T group (Table 5). No deaths occurred during the study, and there were no drug-related serious adverse events. The proportion of patients who prematurely discontinued from the study due to adverse events was 1.8% in each treatment group. Changes from baseline to final visit in several important clinical chemistry, hematology and hemodynamic parameters of interest in men treated with T are summarized in Table 6.

**Table 5.** Treatment-emergent adverse events occurring in >2% of patients in either T treatment group (safety population)

Preferred Term	Oral TU (N = 166)	Topical T (N = 55)
TEAE occurring in >2% of patients, n (%)	78 (47.0)	20 (36.4)
Headache	8 (4.8)	1 (1.8)
Hematocrit increased	8 (4.8)	0
Upper respiratory tract infection	6 (3.6)	0
Hypertension	5 (3.0)	0
High-density lipoprotein decreased	5 (3.0)	0
Nausea	4 (2.4)	0
Rash	2 (1.2)	2 (3.6)
Overdose	1 (0.6)	2 (3.6)

**Table 6.** Percent or absolute change from baseline to final visit in key clinical chemistry, hematology and hemodynamic parameters

Parameter	Oral TU (n)	Topical T (n)
Alanine aminotransferase (U/L)	−5.38 ± 31.72% (159)	−6.09 ± 30.57% (51)
Alkaline phosphatase (U/L)	−14.10 ± 11.84% (162)	−4.83 ± 19.81% (53)
Aspartate amino transferase (U/L)	2.74 ± 39.07% (159)	−0.74 ± 29.64% (51)
Bilirubin (umol/L)	0.41 ± 50.3% (162)	7.8 ± 41.7% (53)
Total cholesterol (mmol/L)	−4.29 ± 15.28% (156)	−5.14 ± 12.13% (51)
HDL_c_ (mmol/L)	−13.91 ± 15.67% (162)	−3.39 ± 16.06% (53)
LDL_c_ (mmol/L)	5.95 ± 26.04% (162)	−2.14 ± 17.71% (53)
Triglycerides (mmol/L)	12.99 ± 46.23% (162)	8.07 ± 35.08% (53)
Prostate specific antigen (ug/L)	0.17 ± 0.48 (161)	0.26 ± 0.38 (51)
Hematocrit (L/L)	5.97 ± 7.46% (160)	4.73 ± 6.65% (53)
Systolic BP		
Cuff (mmHg)	2.8 ± 11.8 (162)	1.8 ± 10.8 (54)
ABPM (mmHg)	4.9 ± 8.7 (162)	0.2 ± 9.4 (45)
Diastolic BP (mmHg)	0.6 ± 8.3 (162)	0.6 ± 7.4 (54)
Heart rate (beats/min)	2.1 ± 9.1 (162)	2.1 ± 7.9 (54)

Investigator-reported TEAEs which occurred more frequently in oral TU patients than in the topical T group were increased hematocrit, hypertension, and decreased high-density lipoprotein (HDL). Each of these events (occurring in 3–5% of oral TU patients) was reported as mild or moderate in intensity and none resulted in premature discontinuation from the study. Decreased HDL events occurred at the higher oral TU doses (316 mg and 396 mg BID), whereas events of increased hematocrit and hypertension were not related to TU dose.

As expected, based on the pharmacological actions of T, mean increases from baseline in hematocrit were observed in both treatment groups at each study visit but remained within the normal range in most men (97% oral TU; 100% topical T). Shifts from normal hematocrit values at baseline to above the normal range were observed in 3% of oral TU patients at the final visit, compared with none of the topical T patients.

No clinically significant changes in the liver function tests [i.e., alanine (ALT) and aspartate (AST) aminotransferases, alkaline phosphatase (AP) and bilirubin] were observed in either treatment group (Table 6). Two oral TU patients experienced increases in AST or ALT that were < 2x the upper normal limit (UNL) and one oral TU patient experienced a transient elevation of AST (<2x UNL) that returned to normal during continued oral TU therapy. Changes in lipid profiles were more pronounced among oral TU patients compared with topical T patients. Shifts from normal baseline to below the normal range for HDL were observed in 28.9% of oral TU patients compared with 14.8% of topical T patients at the final visit. Smaller differences between the treatment groups were observed for shifts from normal baseline to above the normal range in total cholesterol (7.8% versus 3.7%) and triglycerides (13.3% versus 9.3%).

Clinic systolic BP (i.e., cuff) increased from baseline to the end of the study (final visit) in both treatment groups (mean ± SD: oral TU, 2.8 ± 11.8 mm Hg; topical T, 1.8 ± 10.8 mm Hg), whereas diastolic blood pressure was essentially unchanged at the final visit for both groups. Censoring measurements collected after addition of or an increase in dosage of antihypertensive medications had little effect on estimates of mean change for clinic BP. The changes from baseline in systolic BP for the treatment groups were considered in the context of the 2017 AHA/ACC BP classifications (Table 7). The baseline BP was higher in the oral TU group as evidenced by the greater percentage of patients with Stage 1 or 2 hypertension in the oral TU group than in the topical T group, 65% vs 42%, respectively. Testosterone treatment induced a shift to higher BP classifications, but shifts to Stage 1 or 2 hypertension were similar in both treatment groups (31% of the oral TU and 32% of the topical T group).

**Table 7.** Effect of oral TU and topical ton shifts in hypertension classification based on 2017 ACC/AHA BP classifications

	Final Study Visit Classification (%)			
Treatment Group Baseline Classification^a^	Normal	Elevated	Stage 1 HTN	Stage 2 HTN
Oral TU, N = 162^b^				
Normal (22%)	12	5	4	1
Elevated (13%)	3	3	4	3
Stage 1 HTN (51%)	5	6	21	19
Stage 2 HTN (14%)	1	4	5	4
Topical T, N = 54^b^				
Normal (41%)	13	3	11	2
Elevated (13%)	2	4	6	2
Stage 1 HTN (37%)	6	4	17	11
Stage 2 HTN (3%)	2	2	0	6

Abbreviations: dBP, diastolic blood pressure; sBP, systolic blood pressure.

^a^Normal sBP <120 mm Hg and dBP <80 mm Hg; elevated sBP 120–129 mm Hg and dBP <80 mmHg; Stage 1 sBP 130–139 mm Hg or dBP 80–89 mm Hg; and Stage 2 sBP ≥140 or dBP ≥ 90 mmHg.

^b^Four patients in the JATENZO group and 1 subject in Axiron group did not have follow up blood pressure.

Measurement of BP with ABPM yielded greater mean increases from baseline to end of study (approximately 2 days prior to final PK visit) in average daytime (*P* < 0.01), nighttime (*P* < 0.01), and 24-hour (*P* < 0.002) systolic BP for the oral TU group than for the topical T group. The 24-hour average systolic BP increased 4.9 ± 8.7 mm Hg in the oral TU group and 0.2 ± 9.4 mm Hg in the topical T group (*P* = 0.0013). Mean increases in the average daytime, nighttime, and 24-hour diastolic BP from baseline to Visit 6 for the oral TU group were also greater than for the topical T group but the differences were not statistically significant. Among the oral TU patients, mean increases in systolic BP were slightly greater in patients with a history of hypertension who were receiving antihypertensive medication (5.5 ± 8.9 (SD) mm Hg) compared to those with no history of hypertension (4.3 ± 8.6 (SD) mm Hg). There were no discontinuations of oral TU due to hypertension; 5.9% of oral TU patients initiated antihypertensive medication or required a dose increase of existing therapy. The 24-hour average heart rate in the oral TU treated group increased by 2.2 ± 7.7 (SD) beats/minute while in the topical T treated group heart rate decreased by 0.1 ± 6.3 bpm. The changes in heart rate were not statistically significant in either treatment group.

Mean and median changes in PSA were small and comparable between the treatment groups. In addition, a statistically significant difference was not observed between the treatment groups for the proportion of patients with PSA values > 4 ng/mL or with a change from baseline > 1.4 ng/mL at the end of study (1.9% oral TU; 3.8% topical T). Consistent with the PSA data, only minimal and clinically insignificant effects were observed in both treatment groups for urinary symptoms measured by I-PSS.

The cosyntropin stimulation sub-study was included to evaluate the effect of oral TU on cortisol production due to findings of adrenal cortex atrophy and reduced cortisol levels in dogs treated with high doses of oral TU. Although the oral TU group had a significantly lower proportion of patients who had a post-cosyntropin stimulation cortisol value ≥ 18 µg/dL than the topical T group (79% v. 100%), the differences between Day 1 and final study day for maximum cortisol concentrations post-injection and changes in cortisol concentrations from pre-injection showed no statistically significant differences between the treatment groups. Four oral TU patients had cortisol values after cosyntropin stimulation that were slightly below the response cutoff level of 18 µg/dL; however, these responses in cortisol levels (range: 16.5 to 17.6 µg/dL) were not consistent with adrenal insufficiency and not considered clinically significant. Notably, during T treatments (and similar to SHBG levels), cortisol binding globulin levels were suppressed in the oral TU group compared to the topical T group such that when free cortisol levels were calculated, there was no difference the two groups. Only 2 patients had post-stimulation free-cortisol values that were minimally below the published threshold for free cortisol of 1.19 µg/dL (25).

## Discussion

This multicenter, Phase 3 study in adult hypogonadal men was designed to evaluate the efficacy and safety of oral TU to support U. S. regulatory approval. The primary goal of T therapy in hypogonadal men is to achieve mean daily T levels the eugonadal range, avoid excessive peak concentrations (26), and ameliorate symptoms associated with T deficiency. Oral TU restored T to eugonadal levels in 87.3% of patients with an approximate mean serum T C _avg_ of 489 ± 155 ng/dL (converted from NaF-EDTA plasma T values) and with a mean C_max_ response that was in close alignment with FDA targets established for TRT products. In addition, oral TU was associated with clinically significant improvements in symptom relief. The overall safety profile of oral TU was like that for other approved TRT products and reflected the well-recognized adverse effect profile of TRT products as a class (e.g., decreased HDL, increased hematocrit). However, oral TU was associated with a modest mean increase in systolic blood pressure of 3 5 mmHg. As expected, patients treated with oral TU experienced a greater number of GI-associated side effects [e.g., nausea, diarrhea, and burping (often described as ‘minty burps’ owing to the presence of peppermint oil in the oral TU formulation] compared to topical T patients but these were transient, minor in severity and did not result in any patients discontinuing oral TU.

Dose titrations were based on a subject’s total T C_avg_ determined from serial PK sampling over a 24-hour period to ensure a the most accurate characterization of a patient’s T concentration (27). However, dose titrations based on 24-hour sampling are not practical in clinical medicine and thus PK analyses were conducted to demonstrate that a single sample drawn between 4 and 6 hours after the AM oral TU dose could effectively guide dose titration. Total concordance for dose titration between the 4 (C_4_) and 6 (C_6_) hour T concentrations after oral TU administration and C_avg_ was exceptionally strong [C_4_: 88–93%; C_6_: 96–98%] at both titration visits. This indicates that dose-titration decisions based on a single status blood sample drawn from men dosed with oral TU can be used to effectively guide dose titration in a real-world clinical setting.

Although efficacy of TRT therapies in the U. S. is based solely on T PK parameters, the clinical benefit is better assessed using patient reported outcomes such as the PDQ questionnaire. This questionnaire assesses the patient’s sexual health along several domains. In the Testosterone Trials, question 4 of the PDQ questionnaire was one of the primary endpoints and clinically meaningful score change was determined using data from this study (2, 28). In the present study, there were statistically significant improvements in all domains of the questionnaire in both treatment groups. Of particular note, oral TU was associated with a significant increase (both statistically and from a clinical perspective) in the sexual activity domain—a finding consistent with that observed with topical T in this study and in the T trial in older men (28).

Oral TU was associated with a small but significant increase in systolic BP versus topical T. The observed increase in systolic BP in some oral TU patients is consistent with effects reported for a new parenteral (subcutaneous) form of T-enanthate now marketed in the U. S. (29), an older formulation of TU available outside the U. S. [Andriol^®^ (30);] and an oral TU product in phase 3 development [ClinicalTrials.gov identifier: NCT No. 03868059) (31)]. In an effort to determine the etiology of elevated BP we explored the potential relationship between rise in BP to numerous other factors (data not included herein), including: oral TU dose, total and free T, estradiol and DHT concentrations; changes in hematocrit, hemoglobin (as a surrogate for viscosity and increase in plasma volume), potassium (as a surrogate of possible increases in mineralocorticoid levels/activity), and heart rate (as a surrogate of increases in β-adrenergic receptor activity). None of these factors correlated with elevation in systolic BP (R^2^ < 0.04).

The clinical significance of a 3 to 5 mmHg rise in systolic BP in hypogonadal men who have increased cardiovascular risk (32) as result of long-standing T deficiency is unclear. Meta-analyses of large population-based prospective studies demonstrate elevations in BP are directly related to an increased risk of major adverse cardiovascular events including myocardial infarction, strokes and death (33). However, in individual patients, clinically important changes in systolic BP (e.g., confirmed increases > 10 mmHg or resultant values which increase to > 140 mmHg) are detectable by clinicians after initiating treatment with T and can be suitably managed. Importantly, guidelines for the management of hypertension are based on absolute values of the BP and hypertension categories rather than changes in BP (34). During the present study, BP changes in both the oral TU and topical T patients resulted in shifts to higher hypertension categories as evidenced by the fact that the percentage of patients in each treatment group that shifted upwards into Stage 1(130–139/80–89 mmHg) or Stage 2 (≥140/90 mmHg) hypertensive categories was comparable.

Testosterone replacement therapy is known to promote sodium and fluid retention (35), and this may be at least one mechanism leading to elevated BP in some men dosed with oral TU. Regardless of the etiology, careful BP monitoring should be added to the other routine monitoring of men who are receiving oral TU. In addition, men treated with oral TU who have controlled hypertension should be monitored for potential increases in BP that would warrant changes to their hypertension treatment and potential cessation of oral TU.

In prior studies, we have shown that the *ex vivo* conversion of TU to T, as manifested by increases in T concentration after sample collection, was observed in blood samples collected from men receiving oral TU. The rate of conversion was more rapid in the blood samples held at room temperature than kept on ice or collected in tubes that did not contain NaF, a non-specific inhibitor of esterase activity (15). In the present study, T levels used for dose-titration decisions measured in NaF-EDTA plasma to minimize the *ex-vivo* contribution of TU to T measurements. However, in clinical practice, serum is the preferred matrix for T assays. Therefore, we performed a separate study to derive and validate a conversion factor that could be used to convert NaF-EDTA plasma T concentrations in men dosed with oral TU into approximate equivalent serum T concentrations.

Oral delivery of T is simple, convenient and should foster improved patient adherence—a well-described problem with topical T products (36). In addition, oral T administration avoids the potentially painful injection of T-esters (including TU) and eliminates all risk of T transference to women and children, the risk associated with topical gel/solution products. Oral TU was not been associated with liver toxicity in the present study nor in prior clinical studies of this oral TU formulation in which men were treated with oral TU as higher dosages dosed for up to 2 years (ClinTrials.gov: NCT01403116 and NCT01699178). Nor has the new oral TU product been observed to have an overall safety profile different from T replacement products as a class—with the possible exception of effects on BP. However, the magnitude of oral TU effect on BP was the same as that observed for a weekly injected (s.c.) T-enanthate formulation when BP was measured by ABPM (29).

Our study has several strengths and weaknesses. This is the first published study of oral TU in hypogonadal men to demonstrate pharmacokinetic efficacy in line with current U. S. regulatory approval standards. Second, we have demonstrated by two different but related analyses that a single blood sample can be collected about 4–6 hours after the morning oral TU dose to assess the approximate average concentration of T over the dosing interval to reliably guide needed dose adjustments. Third, we have demonstrated that the dose titration scheme prospectively evaluated in this study is robust based on the overall percentage of men who achieved eugonadal T levels and close alignment of peak concentrations with targets established by FDA. Finally, we factored into the study design the potential conversion of TU to T in blood samples collected for T assay in order to generate accurate T measurements on which efficacy was determined. These data, when combined with data from another study (unpublished), enabled us to determine a means by which oral TU response and dose adjustment in a real-world clinical setting can be achieved on the basis of T measured in serum using standard procedures. In contrast to these strengths, studies of this type are not designed to provide outcomes data relative to long-term safety and efficacy parameters and thus, the number of patients evaluated was relatively small. Similarly, the length of oral TU treatment was also fairly brief. Nonetheless, we demonstrated an efficacy and safety profile largely consistent with that observed for a topical T comparator and currently available T replacement products, as a general class.

We conclude that the new oral TU formulation evaluated in this study is a safe and effective means to treat hypogonadal men and has an overall profile consistent with the class of available TRT products but there exists the potential that oral TU may increase systolic BP in some men. The validated dosing and dose adjustment schedule for oral TU should enable treated men to achieve a consistent mean daily T response in the mid-eugonadal range. Oral TU administration is convenient and twice-daily dosing with food (i.e., with breakfast and dinner containing a typical amount of fat) is a simple regimen that may enhance adherence over transdermal and injectable T products that dominate use among hypogonadal men but are associated with pain of administration (injected T-esters) or with transfer of T to women and children.

## Additional Information

***Disclosure Summary:*** TD and RD are employees of Clarus Therapeutics (Clarus) and own stock. RSS, CW, JK, and MG participated as principal and site investigators for the pivotal studies described herein. JK, JAL, and MG have served as consultants for Clarus. CW has received research support from TexoRx and CW and RSS from the NIH for studies on testosterone in older men (T trials). RSS is receiving support from AbbVie as a site for the Traverse Study. RSS and CW have received support from the NICHD, National Institutes of Health, as Centers for Clinical Trials Network Center Director and Project Leader for studies on male contraception where testosterone is one of the drug components. WBW was a safety consultant for Clarus in 2018.

***Data Availability:*** All data generated or analyzed during this study are included in this published article or in the data repositories listed in References.
