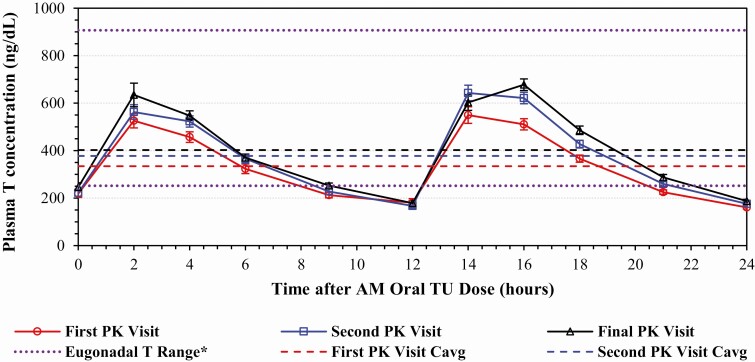# Corrigendum for: “A New Oral Testosterone Undecanoate Formulation Restores Testosterone to Normal Concentrations in Hypogonadal Men”

**DOI:** 10.1210/clinem/dgaa662

**Published:** 2020-10-13

In the above-named article by Swerdloff RS, Wang C, White WB, Kaminetsky J, Gittelman MC, Longstreth JA, Dudley RE, and Danoff TM (*J Clin Endocrinol Metab*. 2020;105(8):2515–2531; doi: 10.1210/clinem/dgaa238), the following transcriptional error occurred in the published paper: “The y-axis title for Figure 3 (page 9) incorrectly describes the plasma testosterone (T) concentration in units of ng/mL instead of ng/dL.”

The authors have provided an updated version of Figure 3.

doi: 10.1210/clinem/dgaa238